# Strategic ejaculation in simultaneously hermaphroditic land snails: more sperm into virgin mates

**DOI:** 10.1186/1471-2148-13-264

**Published:** 2013-12-05

**Authors:** Kazuki Kimura, Satoshi Chiba

**Affiliations:** 1 Department of Environmental Life Sciences, Graduate School of Life Sciences, Tohoku University, Kawauchi 41, Aoba-ku, Sendai 980-8576, Japan

**Keywords:** Sperm competition, Strategic ejaculation, Sperm allocation, Simultaneous hermaphrodites, Land snails

## Abstract

**Background:**

It has been theorised that sperm competition promotes the strategic usage of costly sperm. Although sperm competition is thought to be an important driving force of reproductive traits in simultaneous hermaphrodites as well as in species with separate sexes, empirical studies on strategic ejaculation in simultaneous hermaphrodites are scarce.

**Results:**

In the present study, we tested whether the simultaneously hermaphroditic land snail *Euhadra quaesita* adjusts the number of sperm donated according to the condition of the mate and whether the pattern of strategic ejaculation is in line with previously suggested theories. We found that individuals donated much more sperm when they copulated with a virgin mate than when they copulated with a non-virgin.

**Conclusion:**

The virgin-biased pattern of ejaculation matches the theoretical prediction and suggests that sperm competition significantly influence the reproductive traits of simultaneously hermaphroditic land snails.

## Background

If a female copulates with more than one male and stores the resulting sperm, sexual selection is likely to continue after copulation via sperm competition and cryptic female choice. Although the outcome of sperm competition can be influenced by non-numerical factors such as sperm length and seminal fluid composition [1,2], the relative number of sperm transferred by competing males is a key predictor of fertilization success under post-copulatory reproductive conditions in several species [3-5]. Because sperm cells are relatively small and require less energy to produce than eggs, males are expected to evolve an undiscriminating eagerness to copulate and transfer their sperm, whereas females are expected to show a discriminating passivity [6]. However, accumulating evidence has suggested that sperm and seminal fluids are, in fact, costly to produce [7-9]. Selection would therefore favour males that adjust their ejaculate expenditure according to the indicators of expected fertility in a mating event, which include mate condition and sperm competition levels [10,11]. Indeed, such strategic ejaculation has been reported in many animals, including insects, fish, and mammals [12]. As in species with separate sexes, post-copulatory sexual selection is thought to be an important evolutionary force leading to morphological and behavioral diversification in simultaneous hermaphrodites, which have male and female functions at the same time [13,14]. Moreover, previous empirical studies have shown that such organisms are careful about the use of resources for reproduction and can adjust the allocation of resources between their male and female functions [15]. Therefore, simultaneous hermaphrodites are also expected to be prudent with their limited male reserves. However, the literature on this point has been dominated by studies on species with separate sexes.

Several factors are known to be involved in the strategic usage of limited male reserves. Among these factors, sperm competition risk and intensity have been well studied from both theoretical [16-18] and empirical perspectives [19-21]. The sperm competition risk model considers the probability that sperm competition occurs in a focal female [18]. Alternatively, the sperm competition intensity model applies the number of competitors when females mate with more than two males [17]. For example, animals use the number of rivals and the mating history of their mates as indicators in these cases. Theoretical studies have shown that the properties of the mates with which males should spend more ejaculate are species or population specific [11]. Engqvist & Reinhold [22] have shown that when ejaculates from more than two males compete to fertilize ova (i.e., the sperm competition intensity model), the remating frequency of the mates and the fertilization pattern of the sperm from several mates determine the evolutionary consequences of strategic ejaculation. Their model has predicted that a strategy in which larger ejaculations are invested in favour of non-virgin mates is evolutionary stable whenever the remating rate is low. On the other hand, when the remating rate is high, last-male sperm precedence (i.e., the fertilization priority of sperm from the latter male) is necessary for the evolution of the strategy focusing on non-virgins. Conversely, first-male sperm precedence or random fertilization lead to strategic ejaculation that prioritizes virgin mates. Simultaneous hermaphrodites that have a habit of mating with reciprocal intromission (i.e., they play both male and female roles and exchange sperm in a single mating event) are expected to satisfy the conditions of sperm competition intensity models. Such hermaphroditic animals are thought to be intensely promiscuous because the fertilization success of their male function is expected to increase with an increasing number of matings [23], but see [24], which leads to high remating rates and sperm competition among several individuals. In hermaphrodites, therefore, the strategy of ejaculation should be determined by the sperm priority pattern according to the theoretical models proposed by Engqvist & Reinhold [22]. Contrary to the theoretical prediction, however, the simultaneously hermaphroditic earthworm *Eisenia andrei*, in which the fertilization pattern is thought to be random, exhibits greater ejaculate expenditure during copulation with a non-virgin mate than that with a virgin mate [25].

Other factors have been proposed as important for the allocation of the costly ejaculate of males. Female quality (e.g., fecundity) is thought to influence ejaculate expenditure [26-28]. Moreover, because of their unique reproductive systems, another potential factor affects ejaculate size in simultaneous hermaphrodites. When there is a difference in the expected fitness gain between sex roles, individuals show a preference for a particular sex role, and conflict over the preferred sex role arises [13,29]. Some studies have proposed that such gender conflict could be solved by sperm trading, in which sperm transfer from one mate is conditional upon the other mate’s sperm transfer [30]. When individuals reciprocally intromit their penises in a single mating event, they may alter the amount of sperm transferred to their mates (autosperm) based on the amount of sperm received (allosperm). Although a pattern of strategic ejaculation according to the mate’s mating status (virgin or non-virgin) was reported in a simultaneously hermaphroditic freshwater snail, there remains the possibility that the patterns of resource allocation to a female function differ between virgin and non-virgin mates, and thus the effect of mate’s fecundity was unclear [31]. Moreover, although large individuals of the hermaphroditic land snail *Succinea putris* adjust their ejaculate expenditures according to the body sizes of their mates, this result may be explainable in terms of sperm transfer number of the mates because body size is significantly correlated with ejaculation size in this species [32]. Therefore, experiments that examine these factors at the same time are needed to test the theories of sperm competition intensity in simultaneous hermaphrodites. Although only a limited literature exists on strategic ejaculation in simultaneous hermaphrodites, Baur et al. [33] examined it and found no evidence of strategic ejaculation according to either mate mating status, mate body size, and mate sperm transfer number in the simultaneously hermaphroditic land snail *Arianta arbustorum*. However, it has been suggested that sexual selection including sperm competition is weak in *Arianta arbustorum*[34-36]. Moreover, although Anthes et al. [37] found that copulation duration was correlated with sperm competition intensity in a simultaneously hermaphroditic sea slug, Lange et al. [38] revealed that copulation duration was not a reliable proxy for sperm transfer number in that species. Therefore, whether the pattern of strategic ejaculation in simultaneous hermaphrodites is in line with the theories of sperm competition games remains an unclear, controversial issue.

In this study, we examined the effects of the mating history of the mate (virgin or non-virgin) and other potential factors (quality and sperm transfer of mates) on ejaculate size in the simultaneously hermaphroditic land snail *Euhadra quaesita*. In contrast to the reproductive traits of *A. arbustorum*, those of *E. quaesita* have been suggested to be influenced by sexual selection [39]. In *Cornu aspersum*, it has been determined that allosperm near the internal wall of the allosperm storage organ of a mate can survive longer and may have a better chance of successful fertilisation [40]. Therefore, allosperm from the first donor would reach this limited hospitable location and have priority for fertilization. Indeed, several studies have reported this sperm precedence pattern [41-43], but see [44]. Moreover, species from the Helicoidea including *E. quaesita* and *C. aspersum* have similar basic morphology of genitalia. Therefore, it is expected that *E. quaesita* snail will value virgin mates more highly and donate larger ejaculates to such mates.

## Results

**Figure 1:**
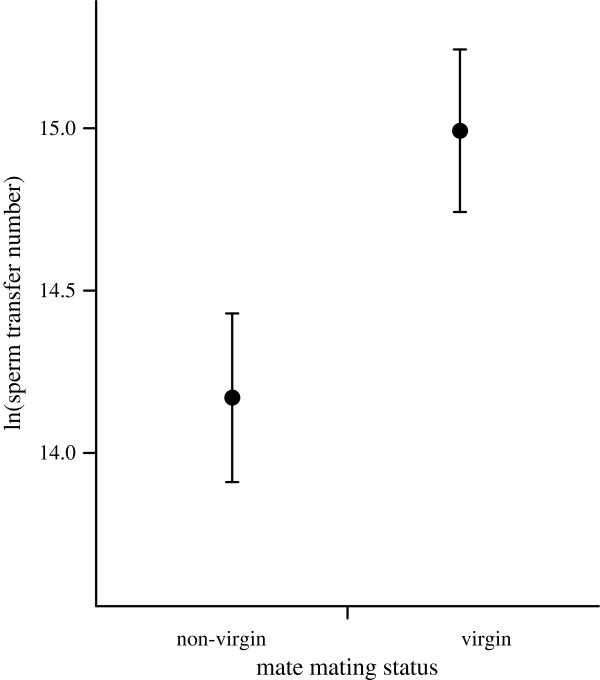
**The numbers of sperm transferred (mean ± SE) to virgin and non-virgin mates.** Sperm transfer number was log (natural) transformed.

We collected spermatophores from 30 pairs of two non-virgin snails and 20 non-virgin / virgin pairs. Although the number of sperm transferred was highly variable (range 1.05 × 10^5^ – 2.11 × 10^7^ for snails in the non-virgin mate treatment; 5.37 × 10^5^ – 1.95 × 10^7^ for the virgin mate treatment) [Additional file 1], similar variabilities have been reported in other species of simultaneously hermaphroditic land snails [33,45,46]. The stepwise deletion of the fixed effects from the linear models produced a model including an effect of mate mating status (Table 1). In contrast, the other two factors associated with the mates (mate body size and mate sperm transfer number) did not affect the number of sperm transferred to the mate. Neither the body size nor the epiphallus length of the snails affected their own sperm transfer. Thus, even when controlling for other mate-associated factors, our model analyses showed that mate mating status significantly affected the number of sperm that a snail transferred to its mate. The snails donated more sperm to virgin mates than to non-virgin mates (Figure 1). On average, the snails that mated with virgins invested 2.20-fold more sperm in the mating (mean = 1.43 × 10^6^ for the non-virgin mate treatment; 3.24 × 10^6^ for the virgin mate treatment).

**Table 1:** Results of stepwise deletion of effects from the initial model for the sperm transfer number

**Variable**	**Coefficient (± SE)**	**Log-likelihood ratio**	**d. f.**	***p*-value**
Intercept	14.17 ± 0.24			
Body size		0.17	1	0.68
Epiphallus length		0.49	1	0.48
**Mate mating status** (A)	**0.82 ± 0.38**	**4.72**	**1**	**0.03**
Mate body size (B)		2.32	1	0.13
Mate sperm transfer Number		1.51	1	0.22
A × B		0.70	1	0.40

Significant effects are in bold.

## Discussion

In this study, we found that the number of sperm transferred by the focal snails differed according to the mating status of the mate in *Euhadra quaesita*. We also found that the pattern of the differences in sperm transfer number was biased toward virgin mates, as was consistent with the prediction of the sperm competition theory under high remating rate and first-male sperm precedence proposed by Engqvist & Reinhold [22]. An explanation other than sperm competition theory, however, may account for this result: if sperm recipients can actively control the transfer of allosperm by the sperm donor, the virgin-biased pattern of ejaculation may be caused by the active influence of the virgin recipients, which do not have any allosperm for cross-fertilization and would have a greater motivation to receive allosperm. However, even if virgins receive the same amount of allosperm as non-virgins, virgins are likely able to fertilize all eggs laid during their lifetimes with this amount of sperm. Moreover, although an excess of allosperm may be used by its recipient as a nutrient source after digestion and absorption, it is also thought to facilitate polyspermy [47,48] and thus lead to decrease in hatched eggs. The risk of polyspermy would be particularly high in the species of the Helicoidea including *E. quaesita* because the donors of these species manipulate the physiology of the recipients and interrupt the digestion of the sperm by the transfer of specific substances [45,49] K. Kimura et al. 2009, unpublished]. Therefore, the difference in allosperm requirement between virgin and non-virgin recipients is unlikely to explain our findings although further experiments are necessary to determine the effect of sperm recipients on the transfer of allosperm. Instead, our results suggested that snails modulate their behavior to use sperm economically in the presence of different sperm competition levels between virgin and non-virgin mates, as expected by sperm competition theory. This likely results in greater fertilization opportunity, although further experiments are needed to confirm this hypothesis. These findings also provided strong support for the prediction that post-copulatory sexual selection has a considerable influence on the evolution of behavioral reproductive traits in simultaneous hermaphrodites [13,23]. It remains unclear which cues snails use to discriminate the mating status of their mates. However, chemosensory cues may play an important role in this discrimination, because it is reported that snails of *E. peliomphala*, a species that is closely related to *E. quaesita*, communicate with air-borne chemicals [50]. Body contact during courtship may also provide an opportunity to communicate and discriminate mating status. How precisely snails can recognize mating status also remains unclear. Engqvist & Reinhold [22] have shown that when animals can discriminate precisely and categorize mates as virgin, singly mated, or multiply mated, a strategy evolves in which animals invest more sperm into singly mated individuals than into virgins. However the relationship between the amount of sperm investment into virgin and multiply mated individuals is approximately same as in a model that assumes only virgin and non-virgin categories (i.e., the model previously discussed). In the present study, we used non-virgin snails that had mated freely in the wild until capture. Because *E. quaesita* is thought to repeat mating in interval of 1–2 weeks [39], the majority of the non-virgins used in this study were most likely multiply mated. Therefore, the facts suggest that regardless of the snails’ ability to discriminate, the virgin-biased pattern found in this study is consistent with the theoretical prediction under high remating rate and first-male sperm precedence by Engqvist & Reinhold [22].

As theoretically predicted for simultaneous hermaphrodites [23], snails of *E. quaesita* exhibit promiscuity and engage in multiple matings between oviposition [51], K. Kimura 2012, unpublished]. Moreover, similar to *Bradybaena fruticum*[52], *E. quaesita* has a simple and unbranched allosperm storage organ, which would not allow sperm recipients to strictly control the use of allosperm stored from several individuals [K. Kimura 2011, unpublished]. Therefore, although further experiments are needed to confirm this hypothesis in our study species, the allosperm of the first mate would occupy a location that is associated with greater survival, as previously shown in *Cornu aspersum*[40], and thus contribute more to fertilization than the last male’s sperm (i.e., first-male sperm precedence). These findings suggest that *E. quaesita* exhibits a high remating rate and sperm priority toward the former donor, which are the conditions required for the evolution of strategic ejaculation in which virgin status is regarded as valuable [22].

Sperm donors are expected to allocate more sperm to more fecund recipients [11,26]. However, we found no evidence that snails adjust sperm transfer number according to the body size of their mates (Table 1). Although the relationship between body size and fecundity in our study species remains unclear, large size tends to be correlated with high fecundity in various species of pulmonate land snails that lay eggs in batches, which would include *E. quaesita*[53]. Factors other than body size may also serve as indicators of fecundity. For example, several studies have reported that males adjust their ejaculation expenditure according to the ages of their mates [54,55]. However, this behavior does not seem to occur in our study species. Virgin individuals of *E. quaesita* are thought to be 2–3 years old, while non-virgins are thought to be 2–6 years old [K. Kimura 2011, unpublished]. In our laboratory experiment, the age variation was expected to be greater in non-virgin mates than in virgins. Therefore, if *E. quaesita* adjusts sperm transfer number according to mate age, sperm transfer number should have varied to a greater degree in the non-virgin mate treatment than in the virgin mate treatment. However, we found that the variations in sperm transfer number were similar between snails that copulated with either mate type (Figure 1). Although further experiments are needed to confirm the effect of mate age, these findings suggest that snails do not behave prudently in terms of mate quality. Our experiment also showed that *E. quaesita* snails select a sperm transfer number independently of the number of sperm received from the mate. Although theoretical studies have proposed that gender conflict between hermaphroditic individuals can be solved by sperm trading [30], the generality of gender conflict and sperm trading is unclear due to a lack of empirical data. Therefore, additional experiments are needed to examine gender conflict in *E. quaesita*. Moreover, how to transfer sperm into a mate may help to understand the independence of sperm transfer number found in this study. *E. quaesita* transfers sperm enclosed in a spermatophore into a mate and this strategy would be against allosperm digestion by the mate [56,57]. Sperm trading has been thought to be associated with the uncertainty of male fertilization success resulting from the allosperm digestion. Therefore, although further experiments are needed to test the hypothesis, such species producing spermatophores may not adopt the strategy of sperm trading.

## Conclusion

In conclusion, our results show that simultaneously hermaphroditic land snails would execute strategic ejaculation according to the mating status of their mates. The pattern of this adjustment was virgin-biased, matching the theoretical prediction. Thus, sperm competition in these land snails strongly influences their behavioral reproductive traits and is more important for snails than the detection of high quality mates.

## Methods

### Study species

We investigated the simultaneously hermaphroditic land snail *Euhadra quaesita* in this study. When individuals of this species reach sexual maturity, they stop their shell growth and form a reflected lip at the shell aperture. *E. quaesita* lives for several years after sexual maturation and undergoes multiple matings. *E. quaesita* forms a spermatophore consisting of a body and a tail. Similar to many other stylommatophorans, the body part of the spermatophore, which is produced in an epiphallus, contains sperm, whereas the tail part, which is produced in the flagellum, does not contain sperm. Although simultaneously hermaphroditic land snails with internal fertilization have divergent reproductive organs [58,59], even within the same species [57,60], the effects of these differences in genitalia have been overlooked in the context of strategic ejaculation in land snails [32,33,61]. In this study, therefore, we investigated the epiphallus length of snails to control for these effects. The mating process of *E. quaesita* consists of courtship behavior and copulation. Two snails that are sexually aroused show courtship behavior, which consists of rubbing on and licking the potential mate. The duration of this courtship behavior is approximately 5–10 min [39]. The snails then simultaneously and reciprocally intromit their penises. The duration of copulation in this species is 100–150 min. During copulation, the spermatophore is introduced into the oviduct of the sperm recipient. Within five minutes of the spermatophore donation, the donor retracts its penis. Although spermatophore donation is not always synchronous, the second donation is usually performed successfully. The sperm then leave the spermatophore and migrate into the allosperm storage organ.

Adult and semi-adult snails were collected in the summer of 2012 from Hachijo Island, Japan and kept individually in plastic pots (450 ml) at 22 degrees Celsius and approximately 65% RH. The snails were fed a powder consisting of barley, protein, and calcium *ad libitum* and their housings were cleaned every 2 weeks. The semi-adults matured within 1–2 weeks after collection and were considered as virgin adults in our laboratory experiments. We categorized the individuals collected as adults as non-virgin snails because a preliminary field investigation had revealed that all adults (N > 20) had already stored allosperm in their bodies. Although the virgin snails were grown under laboratory conditions, their shell sizes did not significantly differ from those of the non-virgins (Welch’s t test, t = 0.62, df = 26, p = 0.54). In addition to mating status (i.e., virgin or non-virgin), three aspects potentially differ between non-virgins and virgins with respect to reproduction. First, the amount of autosperm reserves may be different in non-virgins and virgins because of the matings preceding capture in the non-virgins. Second, the sex allocation pattern (i.e., resource allocation between male and female functions) may also differ, because information on the presence of conspecifics can influence this allocation in simultaneous hermaphrodites [62] and the virgins were isolated before sexual maturation, in contrast with the non-virgins. Third, the age structures may differ: virgins are thought to be 2–3 years old, while non-virgins are thought to be 2–6 years old [K. Kimura 2011, unpublished]. To control the first and second aspects, both non-virgin and virgin snails experienced more than 8 weeks of isolation before the laboratory experiments. Although the interval between matings has been shown to affect the number of transferred sperm [61,63], 8 weeks is likely a sufficient interval to replenish autosperm in *E quaesita*. Because isolation is thought to alter sex allocation [64,65], this period also likely minimise the potential difference in sex allocation patterns between non-virgins and virgins. The impact of age structure is considered above in the Discussion. Moreover, although a decrease in the already stored allosperm would occur over this isolation period, a preliminary investigation has revealed that non-virgins (N = 5) can lay hatchable eggs after one year of isolation. Therefore, sperm competition was expected to occur in non-virgins.

### Experimental procedures

Focal non-virgin snails were selected. These non-virgins were paired with either a non-virgin or a virgin potential mate. Each pair was placed in a small container (2000 ml) and given the opportunity to mate. The individuals of the successfully mated pairs were frozen with liquid nitrogen shortly after mating. The frozen snails were dissected after defrosting. Photographs of the entire reproductive system were taken with a Nikon COOLPIX P7000 camera. The epiphallus lengths of all the snails were measured using Image J software (National Institutes of Health, Bethesda, MD, USA). The spermatophore delivered in the focal mating was pulled out gently. The number of sperm in the spermatophore was counted for each mated snail. The sperm counting procedure was performed as described by Locher & Baur [66], except that the duration and strength of sonication was changed, and DNA staining was not performed. A preliminary investigation revealed that our sonication strength (38 kHz) did not destroy the head of the sperm even after 80 hours of sonication and that the sperm head could be clearly identified using light microscopy due to its large size. A 30 μl sample of the obtained sperm suspension was transferred to a counting chamber (MATSUNAMI MPC-200). The sperm heads in 10 randomly selected cells (1 cell = 25 nl) were counted, and the mean number of heads was used to calculate the total number of sperm in the spermatophore. To evaluate body size, the shell diameter and height of all snails were measured using a vernier caliper.

### Statistical analyses

Linear models were used to test whether sperm transfer number was affected by body size, epiphallus length, mate body size, mate sperm transfer number, or mate mating status (virgin or non-virgin). Both body size (shell diameter × shell diameter × shell height) and epiphallus length were log (natural) transformed prior to the linear model analyses. Sperm transfer number was also log (natural) transformed because this transformation supported the assumption of a Gaussian distribution (Kolmogorov-Smirnov test, *D* = 0.11, *p* = 0.53). In the linear models, the number of sperm transferred by the non-virgins was treated as a dependent variable. Body size, epiphallus length, mate body size, mate sperm transfer number, and mate mating status were treated as fixed effects. The two-way interaction between mate body size and mate mating status was also included in the linear models as a fixed effect. The significance of the fixed effects in the linear models was assessed with an analysis of deviance (likelihood ratio tests using a chi-square approximation). The models were simplified by the stepwise deletion of non-significant (*p* > 0.05) fixed effects, beginning with the effect showing the highest *p*-value [67]. The linear model analyses were conducted in R 2.15.1 [68].

### Availability of supporting data

The data set supporting the results of this article is included within the article and its additional file.

## Competing interests

The authors declare that they have no competing interests.

## Authors’ contributions

KK designed the study, performed the experiments, analyzed the data, and drafted the manuscript. SC participated in the design of the study and drafted the manuscript. Both authors read and approved the final manuscript.

## Supplementary Material

Additional file 1 Sperm transfer number of focal snails. Click here for file
